# Exploring Transport Behavior in Hybrid Perovskites Solar Cells via Machine Learning Analysis of Environmental‐Dependent Impedance Spectroscopy

**DOI:** 10.1002/advs.202002510

**Published:** 2021-06-21

**Authors:** Dohyung Kim, Eric S. Muckley, Nicole Creange, Ting Hei Wan, Myung Hyun Ann, Emanuele Quattrocchi, Rama K. Vasudevan, Jong H. Kim, Francesco Ciucci, Ilia N. Ivanov, Sergei V. Kalinin, Mahshid Ahmadi

**Affiliations:** ^1^ Joint Institute for Advanced Materials, Department of Materials Science and Engineering University of Tennessee Knoxville TN 37996 USA; ^2^ The Center for Nanophase Materials Sciences Oak Ridge National Laboratory Oak Ridge TN 37831 USA; ^3^ Department of Materials Science and Engineering North Carolina State University Raleigh NC 27606 USA; ^4^ Department of Mechanical and Aerospace Engineering The Hong Kong University of Science and Technology Hong Kong; ^5^ Department of Molecular Science and Technology Ajou University Suwon 16499 Republic of Korea; ^6^ Department of Chemical and Biomolecular Engineering The Hong Kong University of Science and Technology Hong Kong

**Keywords:** distribution of relaxation time, hybrid perovskites, impedance spectroscopy, machine learning, solar cells

## Abstract

Hybrid organic–inorganic perovskites are one of the promising candidates for the next‐generation semiconductors due to their superlative optoelectronic properties. However, one of the limiting factors for potential applications is their chemical and structural instability in different environments. Herein, the stability of (FAPbI_3_)_0.85_(MAPbBr_3_)_0.15_ perovskite solar cell is explored in different atmospheres using impedance spectroscopy. An equivalent circuit model and distribution of relaxation times (DRTs) are used to effectively analyze impedance spectra. DRT is further analyzed via machine learning workflow based on the non‐negative matrix factorization of reconstructed relaxation time spectra. This exploration provides the interplay of charge transport dynamics and recombination processes under environment stimuli and illumination. The results reveal that in the dark, oxygen atmosphere induces an increased hole concentration with less ionic character while ionic motion is dominant under ambient air. Under 1 Sun illumination, the environment‐dependent impedance responses show a more striking effect compared with dark conditions. In this case, the increased transport resistance observed under oxygen atmosphere in equivalent circuit analysis arises due to interruption of photogenerated hole carriers. The results not only shed light on elucidating transport mechanisms of perovskite solar cells in different environments but also offer an effective interpretation of impedance responses.

## Graphical abstract

The stability of (FAPbI_3_)_0.85_(MAPbBr_3_)_0.15_ perovskite solar cell is explored in different atmospheres using impedance spectroscopy. An equivalent circuit model and distribution of relaxation times are used to analyze the impedance spectra supported by an unsupervised machine learning method. The transport behavior at the interface and bulk is strongly dependent on the environmental conditions particularly under 1 Sun illumination.

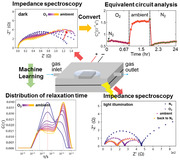

## 1 Introduction

Over the last few years, hybrid organic–inorganic perovskites (HOIPs) have emerged as excellent next‐generation semiconductors due to their outstanding optoelectronic characteristics,^[^ ^1^, ^2^, ^3^ ^]^ facile solution processing, and inexpensive production cost. These qualities have made HOIPs attractive for applications in photovoltaics,^[^ ^4^ ^]^ photodetectors,^[^ ^5^ ^]^ light‐emitting diodes (LEDs),^[^ ^6^ ^]^ and high energy radiation sensors.^[^ ^7^ ^]^ In particular, rapid improvement in the performance of perovskite solar cells (PSCs) has resulted in power conversion efficiency (PCE) exceeding 25.2%.^[^ ^8^ ^]^ However, the key roadblocks to their broad deployment in applications are their chemical instability and environmental sensitivity due to complex defect states and sets of possible electrochemical interactions.^[^ ^1^, ^9^ ^]^ These defects can allow the infiltration of either nitrogen, oxygen, or water molecules from the external environment to passivate the film surface or to accelerate degradation of HOIP devices.^[^ ^10^ ^]^ This necessitates fundamental understanding and precise control over the evolution of ionic and electronic transport sensitivity to the environmental interactions as it has a critical impact on the utility of HOIP devices for future optoelectronic applications.

Several researchers have demonstrated that the interplay between HOIPs and gaseous environments has a significant influence on optoelectronic properties and photostability. For example, it has been shown that nitrogen soaking not only assists stabilization of uncoordinated Pb^2+^ species and organic cations on the surface of these materials but also provides a positive effect on the hole transport.^[^ ^11^ ^]^ Oxygen molecules play an important role in filling charge traps^[^ ^12^ ^]^ and affect lattice strain and electronic states.^[^ ^13^, ^14^ ^]^ Upon oxygen pressure variation, electronic and ionic conduction can be changed because oxygen acts as a substitutional or interstitial acceptor dopant.^[^ ^15^ ^]^ It was also found that water molecules increase ionic conductivity in HOIP thin films.^[^ ^16^ ^]^ On the other hand, combining light and atmospheric treatments has been shown to drastically increase the internal luminescence quantum efficiency of polycrystalline perovskite films, and make the properties comparable with single‐crystalline counterpart.^[^ ^17^ ^]^ It was proposed that light generates superoxide species from O_2_ which reduces the density of traps. Recently, we have shown that the electronic and ionic transport in single crystals of HOIP have a strong environmental dependence, especially at the interface between electrodes and crystals.^[^ ^18^ ^]^

Although it is well established that MAPbI_3_‐based solar cells experience rapid degradation when exposed to both light and oxygen,^[^ ^19^ ^]^ compositionally engineered perovskites, e.g., MAPb(I_0.83_Br_0.17_)_3_, FA_0.83_MA_0.17_Pb(I_0.83_Br_0.17_)_3_, and Cs_0.1_(FA_0.83_MA_0.17_)_0.9_Pb(I_0.83_Br_0.17_)_3_, have shown to effectively suppress degradation rate under illumination.^[^ ^20^ ^]^ The environmental dynamics can contribute to the defect‐induced trap states, resulting in the strong interplay between photogenerated carriers and the crystal lattice.^[^ ^21^ ^]^ Particularly, FA/Cs‐based perovskites have shown better stability than MA‐based perovskites under light and thermal conditions due to a more rigid structure between FA cation and inorganic framework, and smaller Cs ion substitution into organic cation sites.^[^ ^22^, ^23^ ^]^ Several studies have demonstrated triple‐cation perovskites improve the photostability^[^ ^23^ ^]^ and have lower degradation rate^[^ ^24^ ^]^ due to the shrinking of perovskite lattice.^[^ ^20^ ^]^ However, CsI that is effectively used to stabilize the black phase in the mixed (FAMA) cation perovskites is not favorable to the structural stability, especially at high humidity condition (>70% RH) due to its hydrophilic nature.^[^ ^25^ ^]^ Double cations (FAMA) perovskite solar cells have also achieved high efficiencies,^[^ ^26^ ^]^ with significant long‐term stability.^[^ ^27^ ^]^ Despite intensive studies on double cations perovskites, a systematic study on the combined effect of atmospheric molecules and illumination in double cations (FAMA) perovskites is still missing.

Here, we investigate the stability of PSCs with the structure of a fluorine‐doped tin oxide (FTO) glass/SnO_2_/(FAPbI_3_)_0.85_(MAPbBr_3_)_0.15_/Spiro‐OMeTAD/Au using impedance spectroscopy (IS) integrated with an environmental cell to differentiate the impact of the environment and light on the individual electronic/ionic elements including bulk, surface, and interfaces via using equivalent circuit analysis and distribution of relaxation time (DRT). IS has been widely used to study electronic and ionic transport and recombination processes in optoelectronic devices. Despite the widespread application of IS in PSCs, it has been challenging to precisely correlate the physical properties with the individual elements of the circuit model. According to previous studies, the IS of HOIPs shows two distinct responses at low frequency (<1 Hz) and high frequency (>1 MHz) regimes.^[^ ^28^, ^29^, ^30^ ^]^ The faster dynamics at higher frequency regime is associated with recombination of free carriers in an RC circuit model.^[^ ^31^ ^]^ While the slower dynamics at lower frequency regime is related to the ionic motion or accumulated charge carriers at the interfaces.^[^ ^32^ ^]^ When the impedance data is analyzed using an equivalent circuit model, it is critical to select proper individual elements in the model to explain the physical phenomena.^[^ ^33^ ^]^ There has been controversial discussion in addressing the low frequency dynamics in PSCs. Several studies have shown that the slow dynamics are regularized as a capacitor or an RC circuit^[^ ^34^ ^]^ while others consider it as a constant phase,^[^ ^35^, ^36^ ^]^ Warburg (Z_W_),^[^ ^37^ ^]^ or Gerischer (Z_G_) elements.^[^ ^38^ ^]^ While IS provides insight into transport phenomena, making systematic conclusions and decipher associated mechanisms without the material‐ and method‐specific workflows that allow traceable and systematic exploration of the external effects on system response has been absent.

In this study, IS measurements are performed on PSC under different environments in dark and under illumination. An equivalent circuit model is applied to interpret the origin of transport behavior in different environments. To avoid the uncertainties in capacitive components arising from incorrect selection of the circuit model or overfitting, a common mistake, we further analyze impedance spectra by using model‐independent distribution of relaxation time (DRT) method, which is commonly used for batteries and electrochemical reactions, and recently applied to HOIPs.^[^ ^39^, ^40^, ^41^ ^]^ We have recently modified this method by statistical model selection for estimation of the circuit resistances.^[^ ^39^ ^]^ Due to the availability of open‐access databases, machine learning techniques have been rapidly advanced even in the field of perovskites.^[^ ^42^ ^]^ Here, to model the stimulus‐dependent transport behavior in PSCs, we perform a non‐negative matrix factorization (NMF) approach on the DRT data allowing to classify complex signals for better understanding the interplay of charge transport dynamics under environment stimuli and 1 Sun illumination.

## 2 The Effect of Environmental Condition on Impedance Spectroscopy of Perovskite Solar Cells

**Figure 1:**
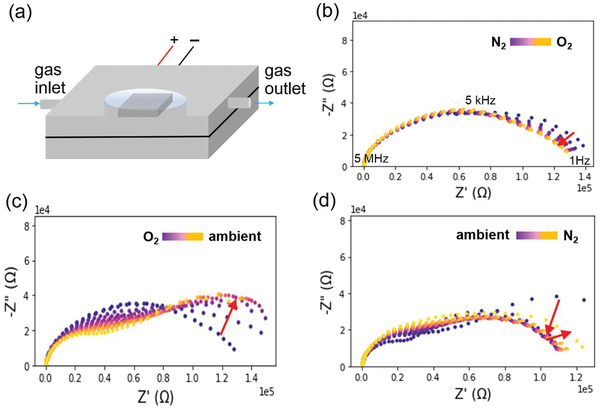
Impedance spectroscopy (IS) of PSC in different environments in dark condition. a) Schematic illustration of IS on a perovskite solar cell sealed inside an environmental chamber. Nyquist plot of frequency dependent IS when b) exchanging the environment from N_2_ to O_2_, c) from O_2_ to ambient condition, and d) back to N_2_ from ambient condition in dark.

**Figure** **1**a shows a schematic illustration of the experimental set‐up for environment dependent IS measurements. The structure of the solar cell is comprised of FTO glass/SnO_2_/(FAPbI_3_)_0.85_(MAPbBr_3_)_0.15_/Spiro‐OMeTAD/Au. Details of sample preparation are described in the Experimental Section. The *J–V* curve and photovoltaic parameters of the fabricated solar cell for this study are shown in Figure S1 (Supporting Information).

To assess the influence of the atmospheric gas on PSCs, the impedance spectra were monitored in different gas environments in dark condition (Figure 1b–d). The IS was performed in the following sequences: dry N_2_, dry O_2_, ambient air with relative humidity (RH) of ≈38%, and back to dry N_2_ (25 °C, 1 atm). Note that dry N_2_ and O_2_ are introduced into gas inlet of the chamber at a constant flow rate. To maintain a constant pressure inside the chamber, an equivalent flow exits from the gas outlet. Ambient air is introduced by opening the chamber after turning off the gas. Samples are exposed to each gas condition for 1 h and the IS response variations are monitored every 2 min. Note that all our results are confirmed through several repetitive measurements. As can be seen in Figure 1b–d, the impedance spectra are strongly dependent on different environments. A significant change in impedance response is observed when the PSCs are exposed to the ambient conditions. To stabilize the PSCs before each gas exposure and measurements, dry N_2_ was flowed through the chamber overnight, recovering the PSC back to the original state as suggested earlier.^[^ ^11^ ^]^ All IS results are presented in the form of Nyquist plots where full impedance *Z*(*ω*) is composed of a real impedance, *R*_e_(*Z*) = *Z* cos *θ*, an imaginary impedance, *I*_m_(*Z*) = *Z* sin *θ* components, and *θ* is the phase shift. Here, all measurements are performed in the frequency range from 5 MHz to 1 Hz at a fixed 100 mV AC bias similar to earlier studies.^[^ ^43^, ^44^ ^]^ Previously, the semicircle in the high frequency regime (HF, close to the origin) has been associated with the electronic transport and recombination processes whereas the response in the low frequency regime (LF) has been attributed to the ionic motion and charge accumulation at the interfaces.^[^ ^30^, ^43^, ^45^, ^46^, ^47^ ^]^

Under dry N_2_ condition, the observed semicircle remains unchanged at all frequency regimes during repetitive measurements. However, at low frequencies, the single semicircle is changed gradually when the device is exposed to the O_2_ environment. The doping effect of oxygen^[^ ^14^, ^15^, ^48^ ^]^ and the dynamic change in the impedance spectra can be seen in Figure 1b, when O_2_ is flowing continuously for 1 h in the chamber. The time dependent impedance values at LF regime (1 Hz) show that the impedance decreases exponentially and then reaches to an equilibrium state after ≈20 min (Figure S2a, Supporting Information). It is noticeable that the semicircle is distorted when the PSCs are exposed to the ambient air, as shown in Figure 1c. The impedance value at 1 Hz (Figure S2b, Supporting Information) shows an abrupt increase from 128 to around 150 kΩ as soon as the device is exposed to the ambient air and then the impedance is gradually decreased to 124 kΩ after 1 h. The initial increase in the impedance could be due to oxygen desorption. To rule out the effect of moisture on the impedance response of our solar cell, we examine the changes in the impedance spectra from N_2_ to O_2_ atmosphere. As can be seen in Figure S3 (Supporting Information), the semicircle shifts to the lower values in both real and imaginary impedances when the device is exposed to O_2_ environment. The semicircles shift back to higher values when returned to the N_2_ environment. However, there is a partial change especially at low frequency regime as the second semicircle becomes more pronounce. Full or permanent degradation is not observed in this experiment. It was reported that distorted semicircle is one of the evidence of device degradation when exposed to high moisture conditions.^[^ ^43^ ^]^ In addition, O_2_ is harmful under illumination due to the formation of superoxides.^[^ ^23^ ^]^ Here, the changes in impedance behavior in dark condition are more likely due to the interplay of charge carriers with defects. O_2_ molecule have similar size to iodide ions and hence can effectively fill iodide vacancies, recovering complete octahedral coordination around Pb ions.^[^ ^49^ ^]^ Therefore, O_2_ molecules passivates under‐coordinate halide defects which act as nonradiative recombination sites, affecting ionic transport in PSCs. Also, it was reported that oxygen‐induced precipitation of PbO can lead to passivation effects, accompanying the reduction of accumulated anion vacancies and Pb–Pb dimers in the perovskite layer.^[^ ^48^ ^]^ Next, the observed gradual decrease in impedance response under air originates from the water absorption, which initiates partial irreversible response of PSC^[^ ^50^, ^51^ ^]^. It is interesting to note that the distorted semicircle returns to its original shape as the cell is exposed to dry N_2_ (Figure 1d). However, it does not completely recover to the original state (see Figure S2c in the Supporting Information) in 24 h. Figure S4 (Supporting Information) shows semicircles at the original condition and after consecutive experiments. A lower impedance pattern is observed in LF regime rather than HF regime. This indicates that the overall resistances of the device are either partially or permanently changed during exposure to the ambient air. The ambient moisture can permeate the Spiro‐OMeTAD layer and diffuse into the perovskite layer.^[^ ^50^ ^]^ This results are in agreement with the improved device performance when perovskite films are fabricated under high humidity^[^ ^50^ ^]^ or for the air (20–40% RH)‐annealed perovskite films.^[^ ^51^ ^]^ Studies have shown that moisture can diffuse along the perovskite/spiro‐OMeTAD interface from the edge of the cell.^[^ ^52^ ^]^ Note that due to dense and continuous structure of Au electrode, moisture diffusion via this layer is not possible, thus it is impervious for moisture to enter the perovskite layer via this route. While, water and O_2_ transport through spiro‐OMeTAD layer is expected as this layer has an amorphous structure.^[^ ^53^ ^]^ Nonetheless, the diffusion rate of water and O_2_ through the spiro‐OMeTAD is obviously slower than the diffusion along the interface between perovskite and spiro‐OMeTAD layers.^[^ ^52^ ^]^

The uncharged form of the spiro‐OMeTAD has a relatively low hole mobility and conductivity while adding a p‐dopant (LiTFSI) plays an important role in enhancing these properties due to the interplay of the lithium ion with spiro‐OMeTAD.^[^ ^54^ ^]^ Here, the residual lithium ions can react with O_2_ and the spiro‐OMeTAD whereas TFSI^−^ can stabilize the oxidized spiro‐OMeTAD.^[^ ^55^ ^]^ Meanwhile, oxidation by LiTFSI is a relatively slow process which needs the oxygen permeation typically from the ambient air.^[^ ^56^ ^]^ The lithium ions do not have a strong impact on redox potential to oxidize the spiro‐OMeTAD.^[^ ^56^ ^]^ It was reported that the spiro‐OMeTAD oxidation is only optically activated.^[^ ^57^ ^]^ Therefore, in dark condition oxidation of spiro‐OMeTAD could be trivial while it provides a crucial key in enhancing the conductivity under illumination in PSCs.

A great deal of environmental studies suggested atmospheric molecules such as N_2_, O_2_, and H_2_O influence PSCs performance as non‐coordinated atoms on the perovskite surface.^[^ ^11^, ^14^, ^15^, ^18^, ^50^, ^51^, ^58^, ^59^, ^60^ ^]^ It has been proposed that nitrogen stabilizes disordered regions in the perovskites such as grain boundaries, surfaces, or interfaces.^[^ ^11^ ^]^ The interaction between N_2_ molecules, organic and Pb cations results in recovering the perovskite structure such as minimizing ion migration and lattice vibration where N_2_ molecules are integrated into the perovskite lattice discontinuities.^[^ ^11^ ^]^ Furthermore, the substitutional and interstitial incorporation of negatively charged O_2_ molecules can increase the charge carrier density by occupying halide vacancies, leading to the well‐passivated p‐doped surfaces.^[^ ^14^, ^15^, ^18^ ^]^ Simultaneously, positively charged halide vacancies filled by oxygen absorption can also affect ionic transport. Finally, the H_2_O molecules which infiltrate to the surface voids, grain edge, or interfaces, annihilate the bonding between MA^+^ or FA^+^ and Pb–I/Br framework,^[^ ^58^ ^]^ however once the water is removed from the perovskite film or PSC, it can result in the recovery.^[^ ^59^ ^]^ Humidity‐induced degradation occurs rapidly when RH reaches 50% while humidity condition in our experiment is below 40% which does not lead to permanent degradation of PSC.^[^ ^61^ ^]^ However, ambient condition with low humidity level could still have an impact on transport dynamics in PSCs as water molecules interrupt perovskite surface and the localized charge carriers.^[^ ^62^ ^]^ It has been theoretically demonstrated that under application of an electric field, few water molecules can be clustered around MA^+^ ions in the presence of trapped charges on perovskite surface.^[^ ^63^ ^]^ This results in local decomposition of MA^+^ due to breaking hydrogen bonds by water molecules.^[^ ^63^ ^]^ In addition, it has been reported that moisture has a significant effect on perovskite crystallization during device fabrication process, revealing that annealing under ambient air results in higher efficiencies.^[^ ^50^, ^51^, ^60^ ^]^

## 3 Equivalent Circuit Analysis

**Figure 2:**
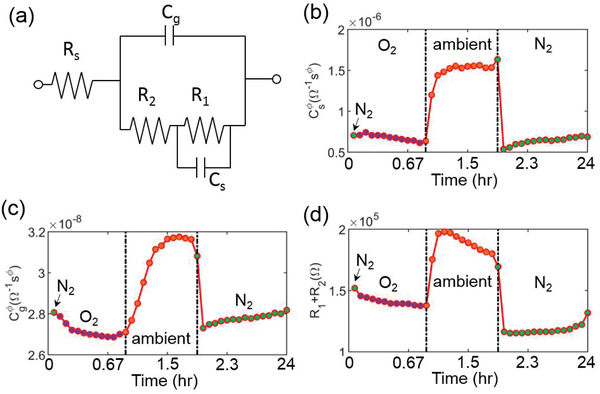
The equivalent circuit analysis of PSC in different environments in the dark. a) Equivalent circuit model comprising two constant phase elements (${\left(i2\pi fC_{s}\right)}^{\phi_{s}}$ and ${\left(i2\pi fC_{g}\right)}^{\phi_{g}}$) and two resistive (*R*_1_ and *R*_2_) elements. Environmental dependence of b) $C_{s}^{\phi_{s}}$, c) $C_{g}^{\phi_{g}}$, and d) *R*_1_ + *R*_2_ as a function of experiment time.

To explore the internal resistive and capacitive response of electronic and ionic mechanisms, we model an equivalent circuit to fit the IS as described in **Figure** **2**a. This model describes underlying relationship between the impedance response and electric/ionic properties at the outer interface as well as the bulk of solar cell.^[^ ^29^, ^30^, ^64^ ^]^ This model is used to explain the internal resistances considering the resistances of the whole PSC. The original equivalent circuit model^[^ ^29^ ^]^ is comprised of two capacitive (*C*_s_ and *C*_g_) and two resistive (*R*_1_ and *R*_2_) elements where *C*_s_ is associated with the surface charge accumulation at the interfaces, and *C*_g_ is related to the dielectric responses in the HOIP layer. Here, we replace the capacitive elements with two constant phase elements (${\left(i2\pi fC_{s}\right)}^{\phi_{s}}\;$and ${\left(i2\pi fC_{g}\right)}^{\phi_{g}}$). The application of constant phase elements allows the improvement in fitting of the IS data and implies a deviation from the ideal capacitive behavior.^[^ ^65^ ^]^ Such nonideality of capacitance is commonly observed in solid‐state systems and can be attributed to nonhomogeneity due to the formation of a double layer at the interface, the roughness of the surface, surface reactions, and the nonuniform current (composition) distribution through the surface.^[^ ^35^ ^]^ Thus, the application of the constant phase element has often been proposed in several studies^[^ ^66^ ^]^ for modeling the impedance of PSCs. The LF regime gives rise to the ${\left(i2\pi fC_{s}\right)}^{\phi_{s}}$ element while the HF regime above 1 kHz contributes to the ${\left(i2\pi fC_{g}\right)}^{\phi_{g}}$ element of the circuit. Our impedance data is fitted well by this model. Standard deviation of all parameters used for this calculation can be found in Figure S5 (Supporting Information). As can be clearly seen from Figure 2a, the shunt resistance *R*_s_, the resistance element that represents the contact ohmic contribution is small and therefore it is neglected in further analysis. With the fitting analysis, the variations in capacitive ($C_{s}^{\phi }$ and $C_{g}^{\phi }$) and resistive (*R*_1_ and *R*_2_) elements in different environments are analyzed in Figure 2b–d. In Figure 2b, with the gradual exposure to O_2_ environment from N_2_, the $C_{g}^{\phi s}$ circuit element that represents the accumulation of ionic charges at the interface in the dark condition^[^ ^67^ ^]^ first decreases from the initial value of 7.0 × 10^−7^ in N_2_ to 6.4 × 10^−7^ Ω^−1^ s*^*ϕ*^* in O_2_. This is possibly due to the reduction in the density of ionic defects in O_2_ environment. For a better visual representation, the area of the curve has been further zoomed in Figure S6a (Supporting Information). However, within first few minutes of exposure to the ambient environment, $C_{s}^{\phi s}$ increases significantly from an initial value of 3.7 × 10^−7^ Ω^−1^s^*ϕ*^ (O_2_ environment) to 1.87 × 10^−6^ Ω^−1^s^*ϕ*^ (ambient air environment) and reaches an equilibrium in 1 h, as shown in Figure 2b and Figure S7a (Supporting Information). Under ambient environment, higher capacitance results in the increased charge carrier density at the interface, corresponding to the accumulation of electronic and ionic charges. The $C_{s}^{\phi s}$ equilibrium is observed within ≈10 min, suggesting that O_2_ gas molecules are physically desorbed within a few minutes as soon as chamber is filled with ambient air. The observed time‐dependent variation of the charge accumulation at the interface has ionic character as the electronic charge carriers lifetime are likely to be below millisecond time scale.^[^ ^41^, ^68^ ^]^ There have been many reports that slow diffusion of ionic defects appears in minutes at room temperature and ambient air.^[^ ^41^, ^47^, ^69^, ^70^ ^]^ Ion migration then in turn is in equilibrium within a few minutes when no additional external stimuli such as bias or illumination are applied. Upon filling the chamber with dry N_2_, $C_{s}^{\phi s}$ dramatically decreases from 1.6 × 10^−6^ to 0.5 × 10^−6^ Ω^−1^ s*^*ϕ*^* in the first few minutes, and then gradually increases during 24 h to nearly the original value, i.e., 0.7 × 10^−6^ Ω^−1^ s*^*ϕ*^* (see Figure S8 in the Supporting Information).

The $C_{g}^{\phi_{g}}$ element of circuit (see Figure 2c and Figure S6b in the Supporting Information) shows substantially less changes of about 0.1 × 10^−8^ Ω^−1^ s*^*ϕ*^* upon transition from N_2_ to O_2_ exposure. The $C_{g}^{\phi_{g}}$ can be attributed to the geometrical capacitance $C_{g}^{\phi }=\;\frac{\varepsilon \varepsilon_{0}}{d}$, where *d* is the perovskite film thickness, *ε* is static dielectric constant of HOIP layer, and *ε*_0_ is the vacuum permittivity. Despite the minor changes, it is reasonable to assume that under ambient conditions the increased $C_{g}^{\phi_{g}}$indicates a small increase of dielectric constant that is directly linked to defect densities as others reported.^[^ ^71^, ^72^ ^]^ O_2_ passivating halide vacancies can lead to the reduction of dielectric constant which is consistent with our result in Figure 2c. Therefore, without O_2_, as ionic defects increase, dielectric constant can be increased under ambient air. Typically, a large dielectric constant plays a role in screening the charged impurities and defects, thereby improving charge transport and recombination^[^ ^71^ ^]^ however, this is not dramatically increased in our experiment. In addition, it takes 30 min for the capacitance to reach an equilibrium, implying halide vacancy‐induced ionic transport rather than electronic charge carrier transport. As observed in our studies, the large change of capacitive responses in different environments results from potential structural variations induced by either distortion of the polar cations^[^ ^73^ ^]^ or ionic movement‐induced structural change.^[^ ^41^, ^74^ ^]^ Another possibility for structural deformation stems from the fact that the charge carriers are strongly localized due to electron–phonon coupling, which leads to the lattice distortion.^[^ ^75^ ^]^

Next, we explore the effect of environment on the resistive elements of circuit, *R*_1 _+ *R*_2_, Figure 2d. According to previous study,^[^ ^29^ ^]^ the recombination resistance in perovskite solar cell depends on the concentration of charged species. Here, *R*_1_ + *R*_2_ which represents the recombination resistance, shows similar behavior as capacitance in different environments. As we change the atmospheric condition gradually from N_2_ to O_2_, the oxygen molecules are absorbed into the perovskite layer. Following the study of defect chemistry by Maier,^[^ ^15^ ^]^ the hole concentration increases in the presence of O_2_ and thereby leads to a reduction in the recombination resistance. As we change the atmospheric condition from O_2_ to the ambient condition, there is a substantial increase in the recombination resistance (Figure 2d). This is consistent with a previous study showing that the existence of moisture leads to an increase in the electron–hole recombination rate, which causes an increase in the recombination resistance.^[^ ^15^ ^]^ However, this circuit element does not fully recover to the initial state when the device is exposed to N_2_ recovering lower recombination resistance state. This suggests higher charge transport than the original state. Our result clarifies that the interplay of ionic motion and charge transport with environmental molecules significantly influences the performance of PSC. Clearly, the ionic motion decreases in O_2_ atmosphere while significantly increases under ambient condition in the device.

## 4 Distribution of Relaxation Time Analysis

To further evaluate the underlying physical mechanisms behind the impedance spectra and the equivalent circuit model we develop the model‐free machine learning based workflow. Here, we use the regularized reconstruction to transform the impedance spectra into the relaxation time distributions, and further explore the stimulus‐induced dynamics of relaxation times via dimensionality reduction methods. As a first step of this analysis, we perform the distribution of relaxation time (DRT) analysis. This method has been previously used by us and others to unravel the time dynamics from the impedance spectra via a statistical selection algorithm.^[^ ^39^, ^41^ ^]^ Theoretically, every distinguishable peak in the DRT spectra represents relaxation time constant, *τ*. As can be seen in the DRT analysis, each environmental condition corresponds to a different number of peaks and peak parameters (position, height, and width).^[^ ^76^ ^]^ In order to extract sparse features and peak decomposition in the DRT analysis we perform multivariate analysis using non‐negative matrix factorization (NMF) method.

**Figure 3:**
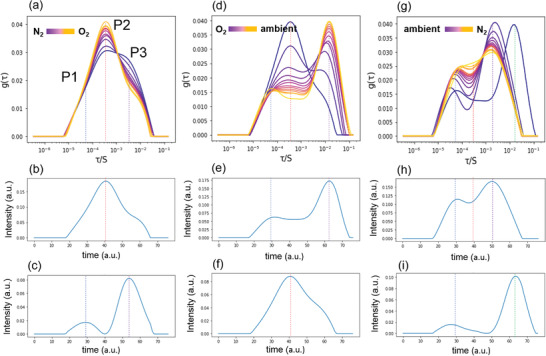
Distribution of relaxation time (DRT) and decomposition of components by nonnegative matrix factorization (NMF) via machine learning. a) Time‐dependent DRT spectra and b,c) two NMF components from the DRT curve when PSC is transferred from N_2_ to O_2_. d) Time‐dependent DRT spectra and e,f) two NMF components from the DRT curve when PSC is transferred from O_2_ to ambient condition from. g) Time‐dependent DRT spectra and h,i) two NMF components from the DRT when PSC is returned to N_2_ from ambient conditions under dark.

The DRT spectra in **Figure** **3**a exhibit approximately three distinguishable peaks (P_1_, P_2_, and P_3_) in the time domains between tens of microseconds to tens of seconds. Here, a negligible change in the intensity and position of the peak (P_1_) is observed. The intensity of peak P_2_ significantly increases while peak P_3_ decreases with O_2_ exposure from the initial N_2_ atmosphere. The peaks P_1_ and P_2_ indicate the faster relaxation time between 10^−5^ and 10^−3^ (s), and peak P_3_ indicates a slower relaxation time around 10^−3^ and 10^−2^ (s), which can be attributed to the slow phenomena such as ionic migration. The decomposition of three overlapped peaks in DRT spectra of Figure 3a is shown in Figure 3b,c.

To get insight into the evolution of relaxation time spectra with the external conditions, they were analyzed using NMF. The role of NMF (multivariate analysis) is to find the common trends in the DRT as a function of external parameters, i.e., environmental condition. In the system containing several circuit elements, the external stimulus can influence on several elements at the same time. The use of the multivariate analysis method will allow us to reveal this effect and elucidate the trend. Here, NMF is chosen as the simplest method that yields non‐negative components, the constrain of relaxation time distributions. In NMF, the stimulus dependence of *G*(*c*, *τ*), where *c* is the index corresponding to experimental and *τ* is time, is represented as

(1) $$G\;\left(c,\tau \right)=\sum_{i\;=\;1}^{N}L_{i}\left(c\right)w_{i}\left(\tau \right)\;$$

where *L_i_* (*c*) are the weights that represent the fraction of response due to specific mechanisms and *w_i_* (*τ*) are the endmembers that determine characteristic behaviors. First, the NMF decomposition of the full data set was explored. The number of components *N* is set at the beginning of the analysis and can be chosen based on the quality of decomposition, anticipated physics of the systems, etc. Here, the endmembers and time dependences for *N* = 3 is shown in Figure S9 (Supporting Information). It is clear that the single peak in component 1 slightly increases from N_2_ to O_2_ atmosphere and rapidly changes in air. Comparatively, second component also changes in air but remains roughly constant in O_2_. Notably, the kinetics of the component's behavior is fairly simple and monotonic in first and second components. However, decomposition in the third component shown in Figure S9d,g (Supporting Information) is more complicated. In this case, analysis reveals the third component starts to show the difference between O_2_ and N_2_ behavior, as seen in ratio of the high and low *τ* peaks. This component represents the difference in O_2_ and N_2_ environmental dynamics. The general trends agree with the analysis of equivalent circuit model. To investigate the individual effect in each condition, we separately performed DRT analysis and NMF decomposition for each gas environment. Here, after experimentation, the *N* = 2 was found to be optimal for the analysis of relaxation time distributions.

The overlapped peaks are well decomposed using NMF method, with a single predominant peak P_2_ followed by P_1_ and P_3_. Under O_2_ environment in the dark condition, the O_2_ molecules slowly diffuse to the PSC, this diffusion takes place over minutes.^[^ ^15^ ^]^ The rate of oxygen permeation into the perovskite layer can depend on the defect density which will be addressed in our future investigations. The oxygen incorporated PSC shows slower P_3_ and faster P_2_ time constants. Senocrate et al. reported that the O_2_ doping under dark conditions depends on its pressure, suggesting an increased electronic conductivity at low pressure, *P*(O_2_) <10^−2^ bar, and an enhanced ionic conductivity at higher pressure, *P*(O_2_) >10^−2^ bar.^[^ ^15^ ^]^ Note that our experimental conditions are at 1 atm >10^−2^ bar. Upon exposure of PSC to ambient air (Figure 3d), the three peaks rapidly change. The peak (P_2_) decreases by about 60% and then completely disappears after 1 h, the P_1_ does not significantly change. The intensity of P_3_ increases with a slight shift in position toward slower relaxation time. This indicates dominating the slow time constant under ambient air Figure 3e due to the impact of ion transport and ion accumulation. When exposed to ambient air, the O_2_ desorption generates more halide vacancies which cause the enhanced ionic transport as shown in Figure 3d,e. It has been demonstrated that exposure to ambient air after deposition of hybrid perovskite film can lead to higher efficiencies.^[^ ^77^ ^]^ Exposure to ambient air has shown to enhance electrical conductivity in perovskite solar cells. This phenomena was associated with oxidation reaction with moisture, resulting in production of both spiro‐MeOTAD^+^·O^2−^ and spiro‐MeOTAD^+^·TFSI^−^ where moisture is necessary to enhance the conductivity in hole transport layer (HTL).^[^ ^78^ ^]^ It was reported that the spiro‐MeOTAD is oxidized to spiro‐MeOTAD^+^·O^2−^ and O_2_ reacts with Li^+^ ions in the presence of LiTFSI generating a stable and more conductive spiro‐MeOTAD^+^·TFSI^−^.^[^ ^54^ ^]^ Both spiro‐MeOTAD^+^·O^2−^ and spiro‐MeOTAD^+^·TFSI^−^ are associated with high conductivities. Another possible reason for the enhanced conductivity is related to ionic transport in perovskite layer.^[^ ^69^ ^]^ Permeated H_2_O and O_2_ molecules into the perovskite layer can interact with defect sites, e.g., halide vacancies. When O_2_ and H_2_O coexist, H^+^ and OH^−^ ions are available for redox reactions or O_2_ desorption.^[^ ^78^ ^]^ The redox reaction can control the conductivity of the HTL due to LiTFSI redistribution across the HTL.^[^ ^78^ ^]^ This effect can be also attributed to the ionic motion as our results show a dominating effect on the slow time constants in DRT analysis when solar cell is exposed to air. When the chamber environment is filled with dry N_2_ again (Figure 3h), the separated peaks converge into a single peak at longer N_2_ exposure. Turning back to the N_2_ environment for 24 h, the DRT spectra return to the initial pattern. The peak (P_3_) is shifted back to a faster relaxation time scale and its intensity gradually decreases, while the P_2_ peak intensity increases. This is likely due to the effect of N_2_ absorption on ionic transport properties in agreement with previous analysis. It is interesting to note from NMF analysis that the P_1_ becomes a main component as shown in Figure 3i compared with the initial condition. Our previous study demonstrated that oxidation under O_2_ leads to reduction of the electron concentration on the perovskite surface, while the increased conductivity in the device is due to an increase of the hole concentration.^[^ ^18^ ^]^ Hence, the decreased P_2_ under ambient air indicates reduction in holes carrier concentration. The increased P_1_ which becomes a main component when the device is returned to N_2_ can be due to an increase in the hole concentration. It was previously demonstrated that O_2_ and N_2_ can slightly p‐dope perovskite surface^[^ ^79^ ^]^ while H_2_O molecules n‐dope perovskite.^[^ ^79^ ^]^ Thus, when return to N_2_ environment, electron/hole carrier concentrations could be altered as seen in Figure 3i. Given that the partial irreversibility is due to exposure to the humidity, water molecules change the perovskite layer into a hydrate phase. Here, a very thin layer on the film surface can be transitioned into an amorphous layer, consisting of lead halide or lead oxide.^[^ ^80^ ^]^ The vacancies existing on the film surface can be eliminated in the presence of these amorphous structures, instead of complete decomposition of the layer, leading to partial recovery of response.^[^ ^17^ ^]^ Thus, we directly observe an improvement of charge carrier transport through a reduction in impedance (see Figure S4 in the Supporting Information) due to eliminated defects after the device is exposed to ambient air for 1 h.

## 5 The Effect of 1 Sun Illumination on IS of PSC in Different Environments

**Figure 4:**
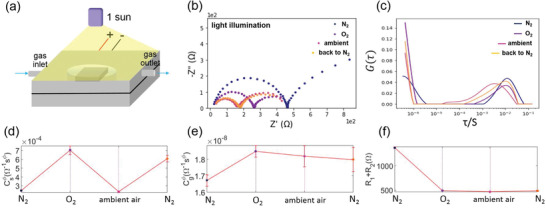
IS of PSC in different environments under 1 Sun illumination. a) Schematic illustration of IS measurement with a perovskite solar cell device sealed inside a chamber under 1 Sun illumination. b) Nyquist plot of PSC under illumination in N_2_, O_2_, ambient air conditions, and back to N_2_. c) Environment‐dependent DRT spectra, and d) $C_{s}^{\phi_{s}},\;e)\;C_{g}^{\phi_{g}}$, and f) *R*_1_ + *R*_2_ variation under 1 Sun illumination.

We further investigated the effect of illumination under controlled environment on the charge transport mechanisms in PSCs, conducting IS measurements under 1 Sun illumination and different environment in the same sequence of gas exposure as in the dark. **Figure** **4**a shows a schematic illustration of the experimental setup for light effects. The PSC was exposed to each environmental condition under continuous illumination for 20 min to stabilize cell performance via light soaking effect.^[^ ^81^ ^]^ Previously, IS under illumination were performed to investigate the interplay of photoexcited charge carrier and ion transport and interfacial effect in PSCs.^[^ ^29^, ^30^, ^44^, ^45^, ^82^ ^]^ The light‐induced ion migration has resulted in several phenomena such as the photoinduced giant switchable photovoltaic effect,^[^ ^83^ ^]^ the photoinstability in perovskite‐based devices,^[^ ^84^ ^]^ and photoinduced phase segregation in mixed perovskites.^[^ ^3^ ^]^ The light obviously accelerates the ionic accumulation due to the increased diffusion coefficient and the reduced activation energy of ions, leading to faster ionic transport under illumination.^[^ ^85^ ^]^ In this study for the first time we explore the interplay of light illumination and environmental gases on transport properties of (FAPbI_3_)_0.85_(MAPbBr_3_)_0.15_ PSC using IS. Figure S10 (Supporting Information) compares the overall impedance response of PSC in the dark and under light illumination. Under illumination, both imaginary and real parts of impedance are dramatically reduced by almost three orders of magnitude due to the dominant effect of photoinduced charge transport, in agreement with previous studies.^[^ ^86^ ^]^

Figure 4b shows that the observed spectra are more sensitive to gas molecules under illumination than in the dark. When the device is illuminated under N_2_ environment, two distinct semicircles in the Nyquist plot are observed similar to the previous reports.^[^ ^30^, ^43^, ^45^, ^46^, ^47^ ^]^ The observed semicircles decrease when PSC is exposed to O_2_. While O_2_ molecules are slowly diffused to PSC in dark, the change under illumination is somewhat faster. Under ambient conditions, the semicircles continuously shrink with a larger semicircle in LF regime. The decreased semicircle at HF regime indicates the reduction of recombination of free carriers while the increased semicircle at LF regime indicating increase in migrated ions and accumulated charge carriers.^[^ ^87^ ^]^ This reveals that the perovskite devices experience an accelerated ionic transport under illumination and ambient air. These results suggest that the combination of light and humidity influences the ionic motion by reducing the activation energy of ionic transport.^[^ ^85^ ^]^ When the atmosphere returns to N_2_ environment, the IS response does not recover to the original state. To further investigate the recovery of PSC, we measured IS response for 70 h in dark (under N_2_) after illumination. As shown in Figure S11 (Supporting Information), the IS responses show similar semicircles, but it does not completely recover to its initial spectrum even after 70 h in the dark under N_2_ environment.

To gain an understanding of the underlying transport mechanisms at each condition, we performed DRT and the equivalent circuit analysis. As shown in Figure 4c from DRT analysis, the peaks on the left side of the time distribution are shifted to the faster time scales due to the effect of photoexcited charge carriers. For the peaks located at microseconds time scale, the highest intensity is seen under the O_2_ atmosphere. Note that the peaks represent the polarization processes and thus peak height reflects the polarization phenomenon.^[^ ^88^ ^]^ The increased polarization is an intrinsic effect associated with increased population of electronic carriers due to a combination of oxygen and light effect. On the other hand, the relaxation time with slower time scales between 10^−3^ and 10^−2^ s related to ion dynamics shows different behavior in different environments. Particularly, under O_2_ atmosphere, the DRT peak shows an asymmetric shape which can be deconvoluted into two peaks with close values of relaxation time. This deconvolution is more prominent under ambient conditions when the slower time constant becomes slightly faster and the second time constant lies between 10^−2^ and 10^−3^ s. The equivalent circuit analysis (all parameters with standard deviation can be found in Figure S12 in the Supporting Information) in Figure 4d–f reveals a dramatic increase in $C_{s}^{\phi }$up to 10^−4^ Ω^−1^ s*^*ϕ*^* under 1 Sun illumination compared with 10^−6^ to 10^−7^ Ω^−1^ s*^*ϕ*^* in dark. This variation in $C_{s}^{\phi }$ results from accumulation of photogenerated charge carriers at the interfaces in agreement with literature.^[^ ^29^ ^]^ Upon O_2_ exposure, $C_{s}^{\phi }$ significantly increases by around 280% from 2.5 × 10^−4^ to 7.0 × 10^−4^ Ω^−1^ s*^*ϕ*^* possibly due to significantly increased charge carrier concentration caused by the reduction of defect density. This leads to accumulation of more charge carriers at the interface between perovskites and transport layers. This phenomenon is contrary to the dark condition. Changing to the ambient condition, the $C_{s}^{\phi }$ is significantly decreased to around 2.3 × 10^−4^ Ω^−1^ s*^*ϕ*^* which is over 300% drop, and then significantly increased by ≈240% to 6.0 × 10^−4^ Ω^−1^ s*^*ϕ*^* in the same order when returning to the N_2_ atmosphere. These results demonstrate that the accumulation of photogenerated charge carriers at the interfaces is significantly dependent on gas atmospheres due to varying defect density especially under light conditions. We have previously shown in MAPbBr_3_ single crystal devices that the environment has a gating effect in the charge transport phenomena especially at the interface between perovskite and the transport layer.^[^ ^18^ ^]^ Meanwhile, $C_{g}^{\phi }$ (see Figure 4e) has similar magnitude compared to that in the dark condition (around 10^−8^), which is consistent with the previous report that the perovskite dielectric response is independent of the light intensity.^[^ ^29^ ^]^ Besides, the *R*_1_ + *R*_2_ element of the circuit displayed in Figure 4f shows a drastic drop in the recombination resistance from N_2_ atmosphere to O_2_ and is almost unchanged in ambient and back to N_2_.

It was proposed that illumination causes lattice expansion,^[^ ^75^ ^]^ phase segregation,^[^ ^3^ ^]^ and ionic movement^[^ ^89^ ^]^ effect in perovskites. There have been also reports on light‐ and oxygen‐induced degradation of PSC.^[^ ^49^, ^90^ ^]^ However, O_2_ related reaction depends on O_2_ partial pressure. In some cases, the reaction between the perovskite layer and O_2_ molecules can lead to decomposition of the perovskite. Such decomposition might originate from moisture as well.^[^ ^15^ ^]^ Recently, dry oxygen‐ and light‐driven degradation of PSCs were observed and explained in terms of the decreased photoinduced hole transport.^[^ ^90^ ^]^ An interruption of hole transport can be derived from the degradation of hole transport layer (HTM) with a reduction of electrical conductivity. Oxygen‐ and light‐induced degradation of HTM can also be due to chemical interaction between two layers, similar mechanism was reported for the device exposed to light and oxygen, and ambient air.^[^ ^90^, ^91^ ^]^ Thus, the hindered hole transport could lead to an increase of charge transport resistance as in our experiments.

Our results have clearly shown the difference in transport behavior of PSC under different environments in dark and under 1 Sun illumination using IS measurements and demonstrate effective analysis of these changes using equivalent circuit model, DRT analysis and NMF signal processing providing successful classification of charge carriers and ionic migration in PSCs.

## 6 Conclusions

We have explored environmental dependence of IS responses in PSCs in dark and under 1 Sun equivalent illumination. The results demonstrate that the impedance spectra are dependent on environments and light. Primarily, the observed single semicircle in the Nyquist plots of a (FAPbI_3_)_0.85_(MAPbBr_3_)_0.15_ based solar cell changes gradually when the device is exposed to the O_2_ environment possibly due to O_2_ doping effects, but the semicircle is distorted as soon as the PSC is exposed to the ambient air. However, the distorted semicircle recovers its original shape upon exposure to dry N_2_ although the recovery is not thorough. We explore the capacitive and resistive elements of equivalent circuit model to understand the transport mechanisms at each condition. Results reveal that the accumulation of ionic charges under O_2_ exposure time decreases due to the reduction of ionic defects, and it increases significantly under ambient air, higher concentration of ionic species. Further, the increased dielectric constant under ambient air indicates the change of defect densities, although the changes are not significant. DRT analysis identifies three different peaks in the time domains between tens of microseconds and tens of seconds. Our results reveal that a slow relaxation time scale corresponding to the ionic migration is increased under ambient air while a fast relaxation time scale corresponding to the electronic transport is increased under O_2_ atmosphere. Here, the variations of peaks in DRT analysis are associated with the interfacial phenomena. Lastly, under 1 Sun illumination, the environmental dependence of IS responses is more significant than the dark condition. When the device is illuminated under N_2_ environment, two distinct semicircles are observed. By exposing device to O_2_, the observed semicircles shrink, and the impedance decreases. Under ambient conditions, the semicircles continuously shrink with a larger semicircle in the LF regime. DRT and the equivalent circuit model confirm that charge carrier transport dominates under oxygen atmosphere whereas ionic transport dominates under ambient air. These studies highlight that the transport behavior at the interface and bulk is strongly dependent on the environmental conditions. The charge transport in PSC results from interplay between defect density and environmental molecule which has a significant effect under illumination. Our findings provide further understanding of transport mechanisms in PSCs based on the exploration of environment‐dependent IS responses and also the deconvolution of IS data via machine learning analysis. These results provide critical information in elucidating transport mechanisms in PSC under different environments and effectively interpret impedance responses using multiple analysis methods.

## 7 Experimental Section

### Solar Cell Fabrication

The fluorine doped Tin Oxide (FTO) were cleaned with deionized (DI) water, acetone, and isopropanol (IPA) in an ultrasonication for 45 min. After drying by the N_2_ gun, an ultraviolet–ozone treatment is carried out for 15 min to remove organic contaminants. The SnO_2_ colloidal precursor (Alfa Aesar) was diluted with DI water to deposit a SnO_2_ layer of 20 nm thickness onto the cleaned FTO glass substrate. Then, it was spin coated onto the FTO substrate with a speed of 3000 rpm for 30 s, and then heated at 150 °C for 10 min after the substrate was transferred to a glove box. The perovskite layer was deposited as follows: MAI(CH_3_NH_3_I) and FAI(NH_2_CH═NH_2_I) were first synthesized with reacting 30 mL hydroiodic acid (57% in water, Aldrich), 27.86 mL CH_3_NH_2_ (40% in methanol, Junsei Chemical), and 15 g formamidine acetate (Aldrich) in a 250 mL round‐bottom flask at 0 °C for 2 h with stirring. The precipitates were obtained using an evaporator at 50 °C for 1 h. The precipitated materials were dissolved in ethanol, recrystallized with diethyl ether, and then dried at 60 °C in a vacuum oven for 24 h. As above, CH_3_NH_3_Br and NH_2_CH═NH_2_Br were also prepared with hydrobromic acid (48 wt% in water, Aldrich). The desired perovskite solutions of (FAPbI_3_)_0.85_(MAPbBr_3_)_0.15_ were spin‐coated onto the SnO_2_/FTO glass. The films were dried on a hot plate at 100 °C for 20 min. After annealing and cooling the perovskite film to room temperature, a 6.5 wt% solution of 2,2′,7,7′‐tetrakis‐(*N*,*N*‐di‐4‐methoxyphenylamino)‐9,9′‐spirobifluorene (spiro‐OMeTAD, Lumtec) including lithium bis(trifluoromethylsyfonyl) imide salt (Li‐TFSI) dissolved in chlorobenzene was coated at spinning speed of 4000 rpm for 30 s. The spiro‐OMeTAD layer was annealed overnight under dry and dark condition to promote oxidation. A gold contact was thermally evaporated onto the spiro‐OMeTAD/(FAPbI_3_)_0.85_(MAPbBr_3_)_0.15_/SnO_2_/FTO glass.

### Impedance Spectroscopy (IS) Characterization

To investigate environment dependence of transport mechanisms, impedance spectroscopy (IS) measurements were performed on the device using a Zahner IM6 electrochemical workstation. To avoid the physical or chemical damage‐induced degradation in the device, Au plated and spring loaded round pogo pins on a PCB substrate were used for reliable electrical contact. The device was sealed inside a chamber (Linkam LTS350). For initial measurement in dark condition, N_2_ gas was purged into the chamber overnight. The impedance spectra was checked constantly and the time‐ and environmental‐dependent impedance responses were measured within 1 h. The data point was captured every 2 min during the measurements. For environmentally dependent transport mechanisms under illumination, the measurements were performed under 1 Sun illumination using a Newport solar simulator. Impedance spectroscopy was recorded inside the sealed chamber under different gaseous conditions at a constant flow rate of around 100 mL min^−1^. The light illumination time was limited to 20 min as continuous illumination causes significantly excessive degradation of the perovskite thin films.

### Equivalent Circuit Analysis

The equivalent circuit model being considered has the analytical expression as follows

(2) $$Z^{\mathrm{eq}}\left(f,\theta \right)=R_{s}\;+{\left({\left(i2\pi fC_{g}\right)}^{\phi_{g}}+{\left({\left(\frac{1}{R_{1}}+{\left(i2\pi fC_{s}\right)}^{\phi_{s}}\right)}^{-1}+R_{2}\right)}^{-1}\right)}^{-1}$$

where ****θ**** is the set of fitting parameters which consists of three parameters from the resistive elements, i.e., *R*_s_, *R*_1_, and *R*_2_, and four parameters from the constant phase element, i.e., *C*_g_, *C*_s_, *φ*_g_, and *φ*_s_. The parameters are estimated by minimizing the sum of squared residual between the measured impedance and the model impedance, i.e.,

(3) $$\hat{\theta }=\underset{\begin{array}{c} 0<\theta ,\\ 0<\phi_{g}\leq 1,\\ 0<\phi_{s}\leq 1 \end{array}}{\mathrm{argmin}}\left[\sum_{m=1}^{M}{\left|Z^{\mathrm{eq}}\left(f_{m},\theta \right)-Z\left(f_{m}\right)\right|}^{2}\right]\;$$

where $\hat{\theta }$ are the set of estimated parameters and *M* is the total number of data points. All fitting for the equivalent circuit analysis was performed with MATLAB.

### Machine Learning: Distribution of Relaxation Time (DRT) and Non‐Negative Matrix Factorization (NMF)

DRT can be mathematically expressed as followed

(4) $$Z\left(f\right)=R_{\infty }+R_{P},\int_{-\infty }^{\infty }\frac{G\left(\tau \right)}{1+i2\pi f\tau }d\mathrm{In}\tau$$

where *Z*(*f*) is the measured impedance, *R*_∞_ is the high‐frequency cutoff resistance, *R*_P_ is the polarization resistance, *f* is the frequencies, and *G*(*τ*) is the density function of relaxation time.

To analyze unmixing components and find multiple archetype profiles from DRT data set, NMF is performed with two components. For NMF analysis, one can represent

(5) X=WH

where *X* = (*n* × *p*), the matrix of *n* samples (rows) and *p* response (columns). *W* (*n* × *k*) and *H* (*k* × *p*) are the scores and loading responses, respectively, and *k* presents the matrix factorization of rank. Here, individual samples are a weighted sum of *k* archetypal profile which can be mathematically expressed as *X* = *WH* + Error term. Notably, factors of *W* and *H* are all non‐negative.

## Conflict of Interest

The authors declare no conflict of interest.

## Supporting information

Supporting Information

## Data Availability

All data used in this manuscript are available from the authors on request.
