# Supplementary material for: Exploring Transport Behavior in Hybrid Perovskites Solar Cells via Machine Learning Analysis of Environmental‐Dependent Impedance Spectroscopy
Source: Adv Sci (Weinh). 2021 Jun 21;8(15):2002510. doi: 10.1002/advs.202002510 (PMC8336513; doi:10.1002/advs.202002510)
Supplement: Supplementary file 1 — Supporting Information [file ADVS-8-2002510-s001.pdf]

© 2021 Wiley-VCH GmbH

ADVANCED SCIENCE
Open Access

Supporting Information

for *Adv. Sci.,* DOI: 10.1002/advs.202002510

Exploring Transport Behavior in Hybrid Perovskites Solar Cells via Machine Learning Analysis of Environmental-dependent Impedance Spectroscopy

*Dohyung Kim, Eric S. Muckley, Nicole Creange, Ting Hei Wan, Myung Hyun Ann, Emanuele Quattrocchi, Rama K. Vasudevan, Jong H. Kim, Francesco Ciucci, Ilia N. Ivanov, Sergei V. Kalinin and Mahshid Ahmadi**

Supporting Information

Exploring Transport Behavior in Hybrid Perovskites Solar Cells via Machine Learning Analysis of Environmental-dependent Impedance Spectroscopy

Dohyung Kim[1], Eric S. Muckley[2], NicoleCreange[3], Ting Hei Wan[4], Myung Hyun Ann[5], Emanuele Quattrocchi[4], Rama K. Vasudevan[2], Jong H. Kim[5], Francesco Ciucci[4,6], Ilia N. Ivanov[2], Sergei V. Kalinin[2] and Mahshid Ahmadi[1*]

[1]Joint Institute for Advanced Materials, Department of Materials Science and Engineering, University of Tennessee, Knoxville, TN 37996, USA

[2]The Center for Nanophase Materials Sciences, Oak Ridge National Laboratory, Oak Ridge, TN 37831, United States, USA

[3]Department of Materials Science and Engineering, North Carolina State University, Raleigh, NC 27606, USA

[4]Department of Mechanical and Aerospace Engineering, The Hong Kong University of Science and Technology, Hong Kong, China

[5]Department of Molecular Science and Technology, Ajou University, Suwon 16499, Republic of Korea

[6]Department of Chemical and Biomolecular Engineering, The Hong Kong University of Science and Technology, Hong Kong, China

*Corresponding author email: mahmadi3@utk.edu

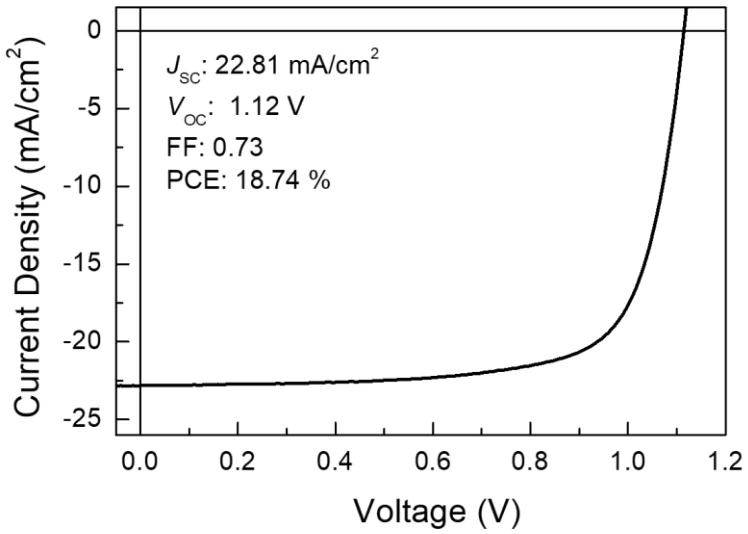

Figure S1. *J*-*V* curve and photovoltaic parameters of $(FAPbI_3)_{0.85}(MAPbBr_3)_{0.15}$perovskite solar cell (PSC) fabricated in this study under one-sun illumination condition.

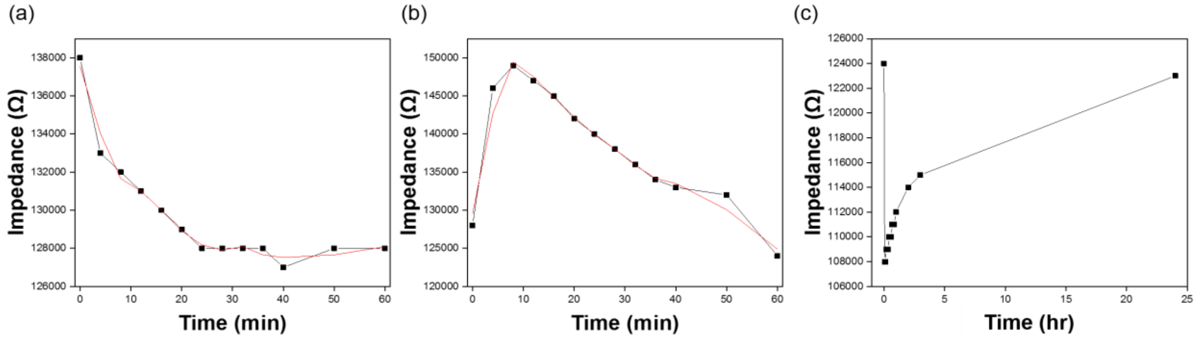

Figure S2. The impedance responseof PSC measured at 1 Hzin dark condition,when (a)the cell is filled from N_2 to O_2, (b) from O_2 to ambient air, and (c) from ambient air to N_2 again.

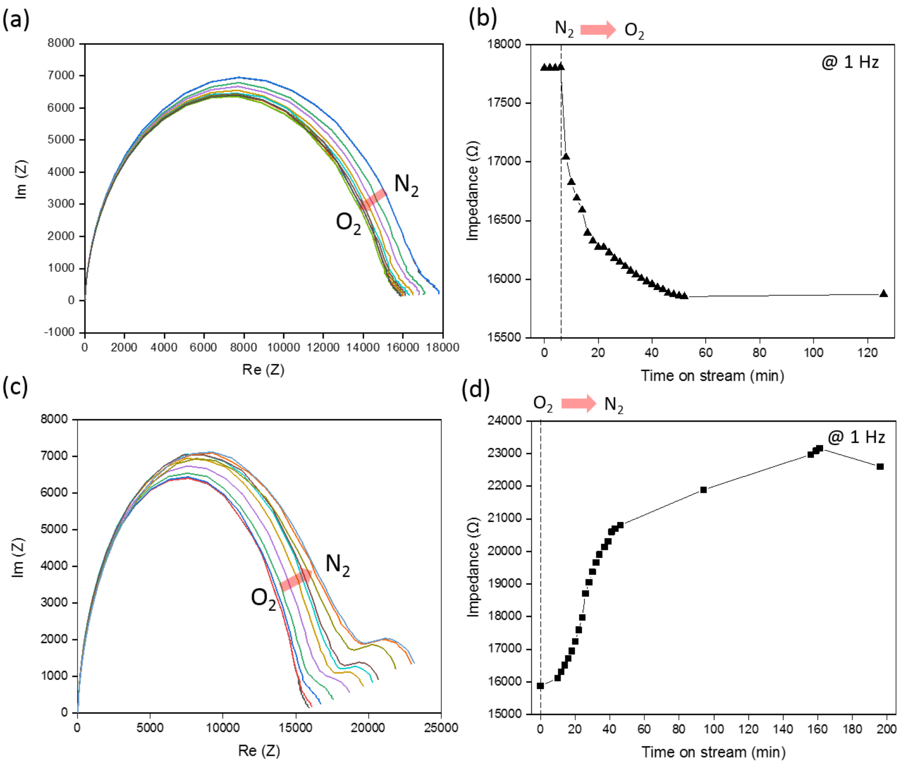

Figure S3. Nyquist plots of impedance spectra and impedance variationsof PSC at 1 Hz from (a-b)N_2 to O_2, and (c-d) from O_2 to N_2 environment, respectively.

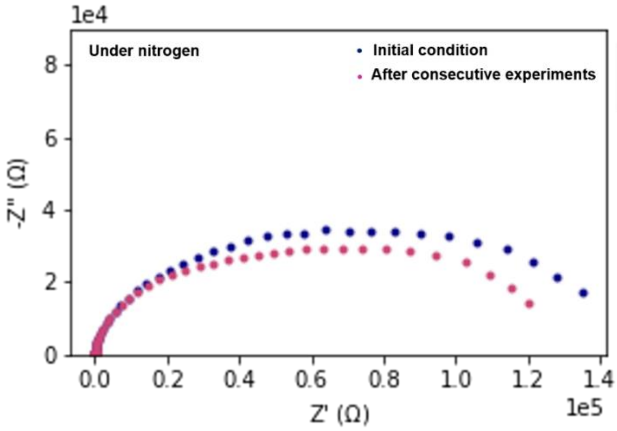

Figure S4. Nyquist plots of impedance spectrafor PSCin dark and under dry N_2atmosphere before and after the consecutive experiments in different environments and back toN_2.

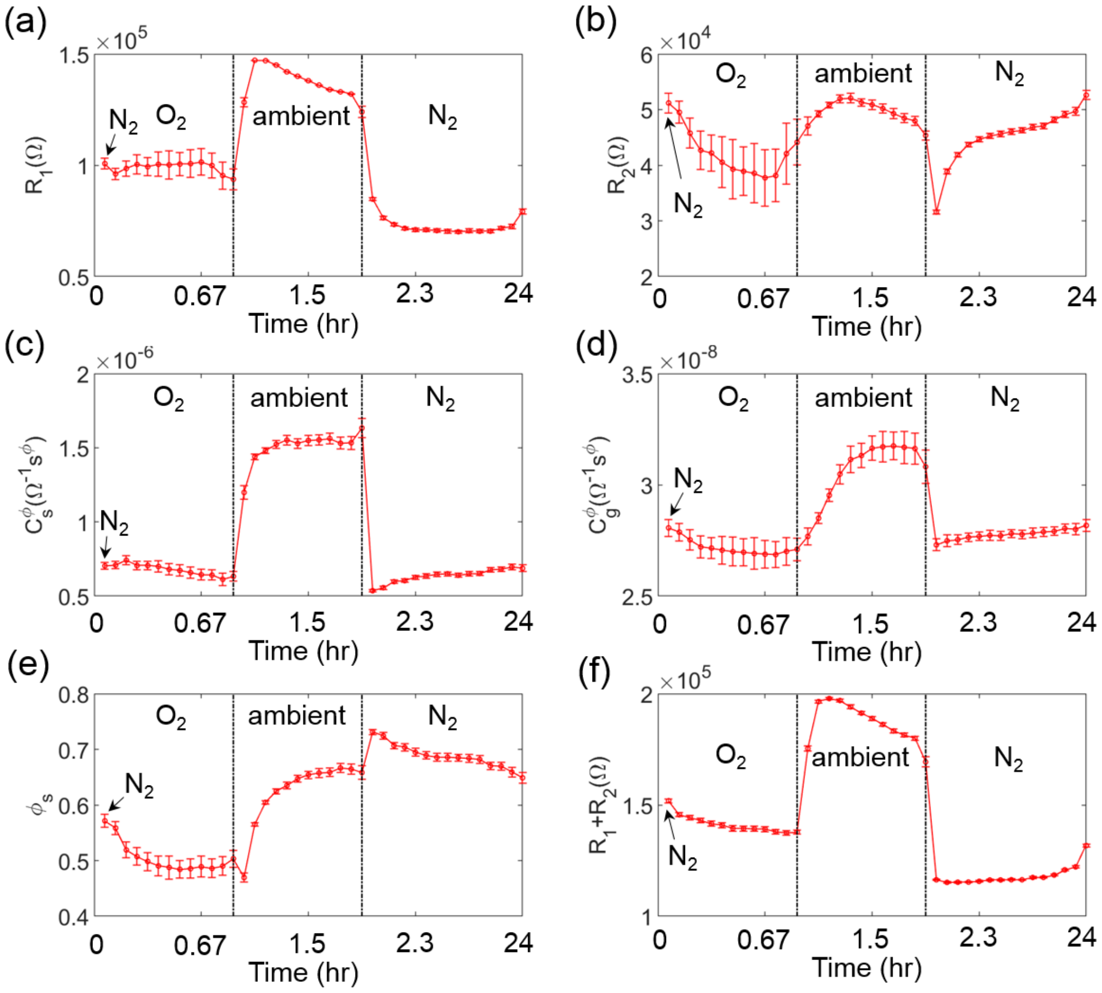

Figure S5. Standard deviation of all parameters in the equivalent circuit analysis of PSC in dark condition. (a) Environmental dependence of R_1, (b) R_2, (c) $C_s^{\phi_s}$, (d) $C_g^{\phi_g}$, (e) ϕ_s, and (f) $R_1 + R_2$ with standard deviation in the dark.

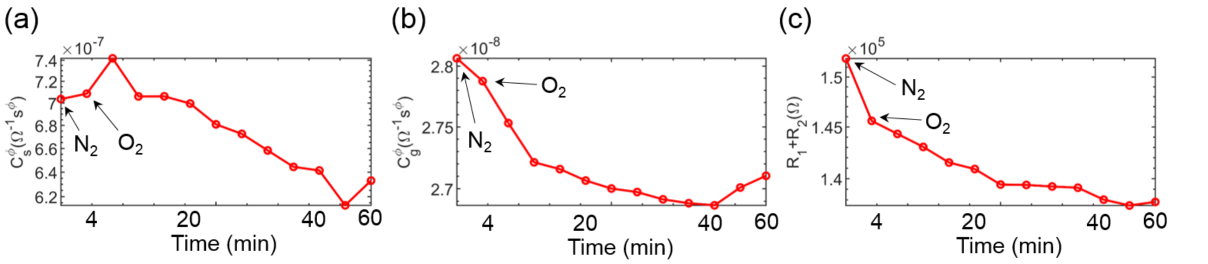

Figure S6. The equivalent circuit analysis when PSC was exposed from $N_2$to O_2atmosphere. (a) Time dependent variation of C_s^{ϕ} , (b) of C_g^{ϕ} , and (c) R_1 andR_2 .

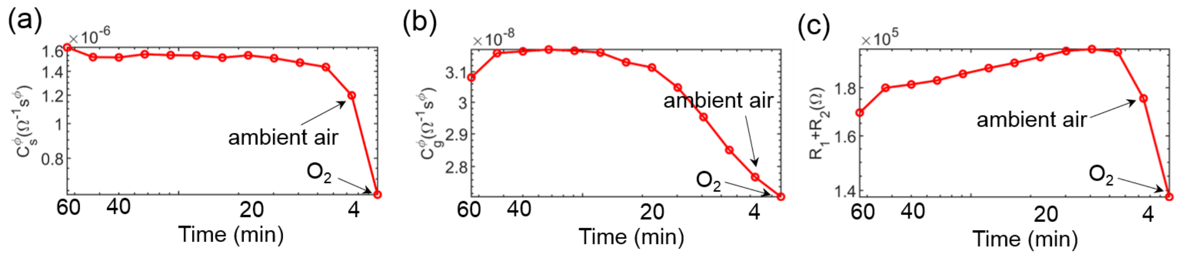

Figure S7. The equivalent circuit analysis when PSCwas exposed from $O_2$to ambient atmosphere. (a) Time dependent variation of C_s^{ϕ} , (b) of C_g^{ϕ} , and (c) R_1 andR_2 .

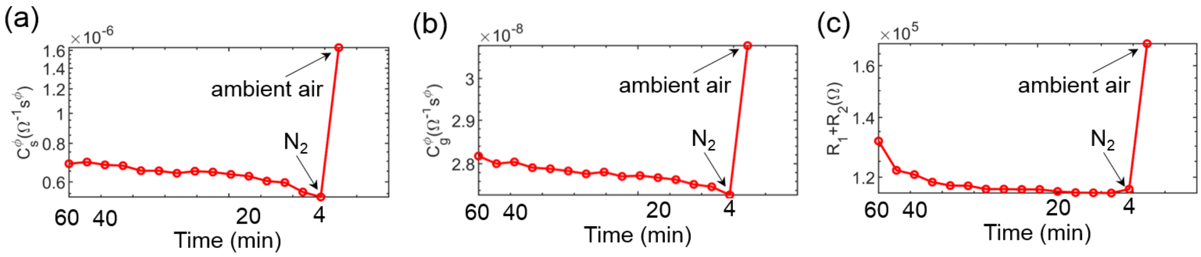

Figure S8. The equivalent circuit analysis when PSC was exposed from ambient to N_2atmosphere. (a) Time dependent variation of C_s^{ϕ} , (b) of C_g^{ϕ} , and (c) R_1 andR_2 .

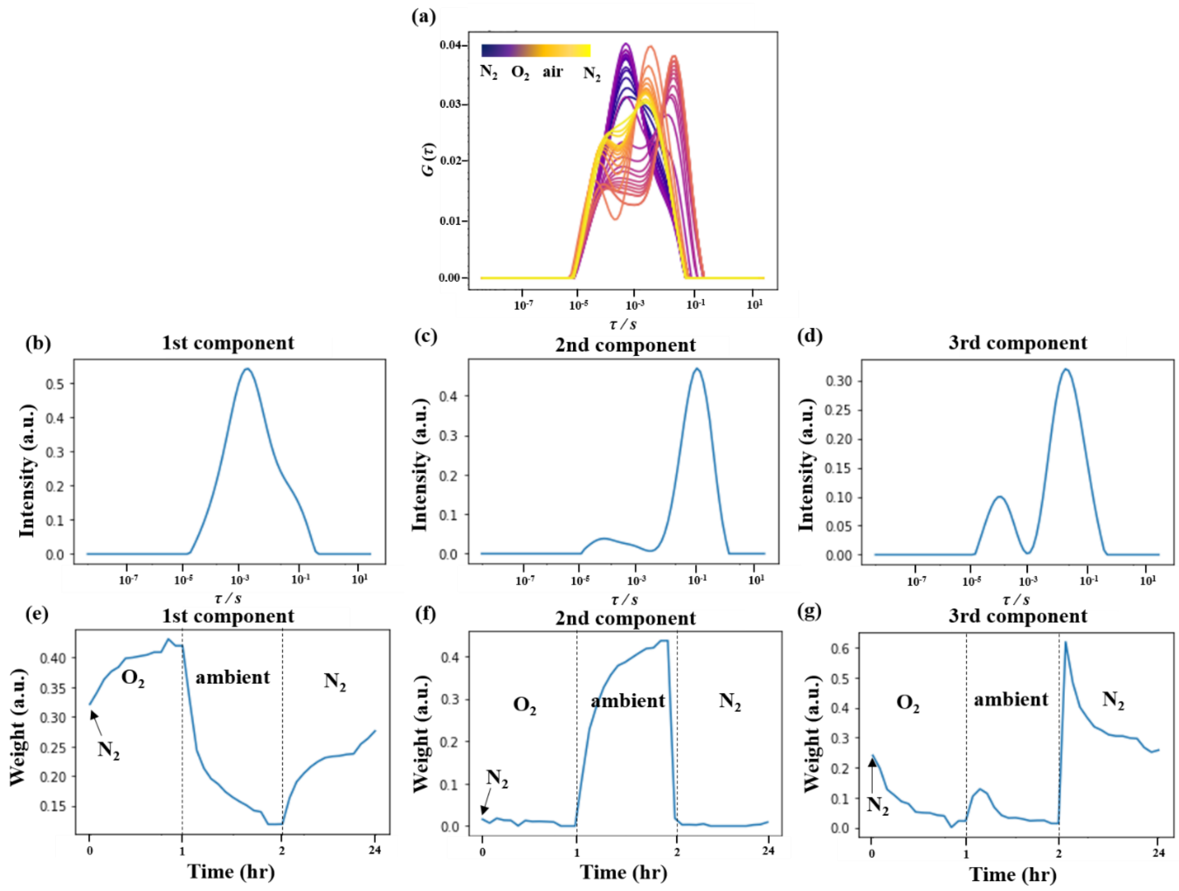

Figure S9. (a) Distribution of relaxation time (DRT) analysis of PSCunder different environments, (b-d) time dependencies of the NMF decomposition of the full data set, (e-g) environmental dependencies of the NMF decomposition of full data set.

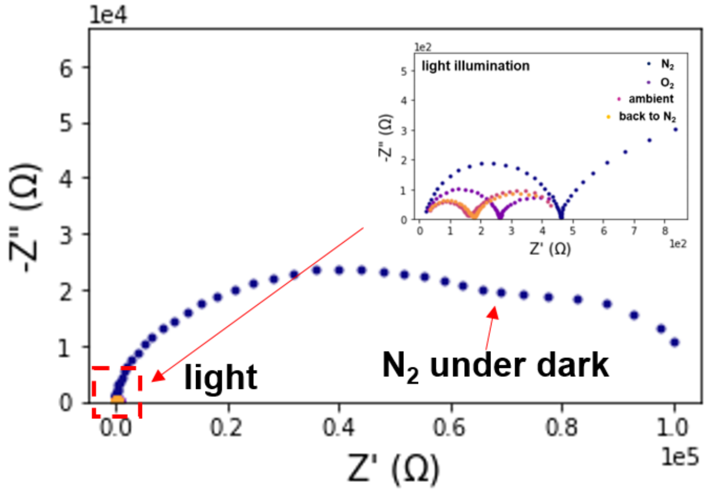

Figure S10. Nyquist plots of impedance spectraof PSC before and after illumination under 1 sun equivalent.

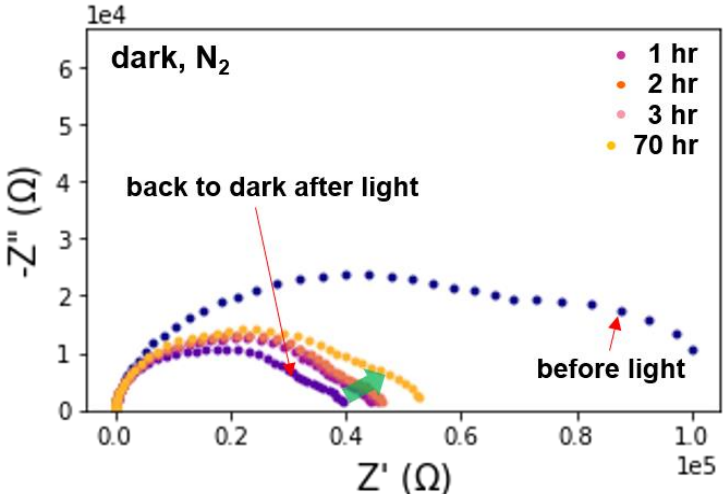

Figure S11. Nyquist plots of impedance spectrain dark after illumination under 1 sun condition. The change inNyquist plotsas a function of time when PSC is back to N_2atmosphere after exposure to different environments underequivalent 1 sun illumination.

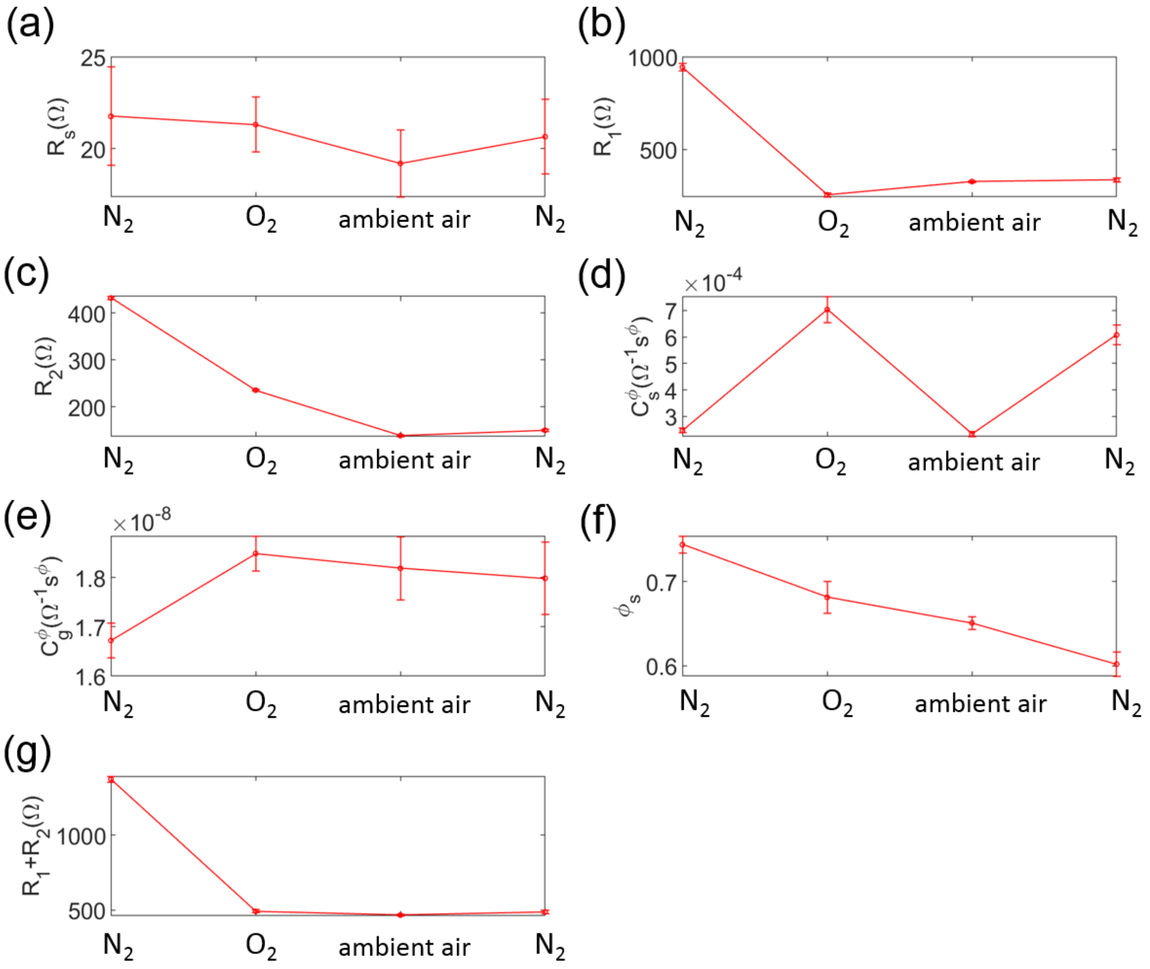

Figure S12. Standard deviation of all parameters in the equivalent circuit analysis. (a) Environmental dependence of R_s, (b) R_1, (c) R_2, (d) $C_s^{\phi_s}$, (e) $C_g^{\phi_g}$, (f) ϕ_s, and (g) $R_1 + R_2$ with standard deviation under 1 sun illumination.
